# Intestinal capillariasis in the 21^st^ century: clinical presentations and role of endoscopy and imaging

**DOI:** 10.1186/s12876-014-0207-9

**Published:** 2014-12-10

**Authors:** Julajak Limsrivilai, Supot Pongprasobchai, Piyaporn Apisarnthanarak, Sathaporn Manatsathit

**Affiliations:** Division of Gastroenterology, Faculty of Medicine, Siriraj Hospital, Mahidol University, Bangkok, Thailand; Division of Diagnostic Radiology, Faculty of Radiology, Siriraj Hospital, Mahidol University, Bangkok, Thailand

**Keywords:** Intestinal capillariasis, Clinical presentation, Enteroscopy, Video capsule endoscopy, Imaging, Treatment

## Abstract

**Background:**

Intestinal capillariasis is one of the common causes of malabsorption in the East. Reports emphasizing the roles of clinical, endoscopic and radiologic findings of intestinal capillariasis are limited.

**Methods:**

Retrospective review of medical records of 26 patients diagnosed with intestinal capillariasis at Siriraj Hospital, Bangkok, Thailand between 2001- 2013.

**Results:**

Clinical manifestations were chronic watery diarrhea (93%), chronic abdominal pain (70%), significant weight loss (92%), hypoalbuminemia (100%; 85% lower than 2.0 g/dL), and anemia (50%). The median duration of symptoms was 5.5 months (1-60 months). Parasites were found in stool in 15 patients (57%). In patients whose stool tests were initially negative, parasites were discovered in tissue biopsy from endoscopy in 1 from 10 esophagogastroduodenoscopies (EGD), 0 from 7 colonoscopies, 3 from 5 push enteroscopies, and 3 from 5 balloon-assisted enteroscopies (BAE). Endoscopic findings included scalloping appearance, mucosal cracking, and redness of mucosa. These endoscopic findings affected mostly at jejunum and proximal ileum. They were similar to celiac disease except duodenal involvement which is uncommon in capillariasis. Three patients underwent video capsule endoscopy (VCE) and typical abnormal findings were observed in all patients. Small bowel barium study showed fold thickening, fold effacement, and increased luminal fluid in 80% of patients, mainly seen at distal jejunum and ileum. CT findings were long segment wall thickening, enhanced wall, and fold effacement. Treatment with either albendazole or ivermectin cured all patients with most responding within 2 months.

**Conclusions:**

In endemic area, intestinal capillariasis should be considered if patients develop chronic watery diarrhea accompanied by significant weight loss and severe hypoalbuminemia. Stool examination had quite low sensitivities in making diagnosis in our study. Deep enteroscopy with biopsy guided by imaging or VCE may improve diagnostic yield. Empirical therapy may also be justifiable due to the very good response rate and less side effects.

## Background

Intestinal capillariasis is an infestation of humans caused by the nematode *Capillaria philippinensis*. The first case was reported in 1963 in Philippines [1], and more than 1,000 patients acquired the illness with over 100 deaths between 1967 and 1969 [2]. Thailand is also an endemic area of intestinal capillariasis with the first case being reported in 1973 [3]. After that, many case reports and case series were published in the literature from different parts of the country [4-9]; the largest was 100 cases from Sisaket province in 1983 [10]. Patients mainly present with chronic diarrhea and severe malabsorption. The diagnosis traditionally is made by discovering the parasitic larvae or eggs in the stool. The standard treatment is albendazole for 14 to 30 days, which achieves full recovery in most patients [11].

Currently, intestinal capillariasis remains problematic in clinical practice. Many patients are difficult to diagnose due to two main reasons. First, the disease has been unrecognized by clinicians. This might be due to atypical manifestations of the disease and the emerging of the disease in non-endemic area [9,12,13]. Second, the sensitivity of stool examination is not high because the parasitic eggs are excreted intermittently in stool. As a result, some patients suffer from symptoms for years. At present, some recently available investigations can help clinicians to examine the affected part of the small bowel more effectively than in the past. Endoscopy such as VCE or BAE have been reported to play role in the diagnosis of capillariasis in a few case reports [14-16]. Conventional imaging such as barium study and advanced imaging such as multidetector computed tomography (MDCT) may also be useful. However, no study has comprehensively described the details of clinical, enteroscopic and radiologic findings in depth. Here, we present a case series of intestinal capillariasis with a detailed description of the clinical presentations, and some investigations including small bowel endoscopic findings and imaging findings which have not been available in other reports.

## Methods

We retrospectively reviewed the medical records, abdominal imaging, and endoscopic findings of 26 patients diagnosed with intestinal capillariasis at Siriraj Hospital, Bangkok, Thailand between January 2001 and June 2013. The present study was approved by the Siriraj Institutional Review Board (SIRB).

The definite diagnosis of capillariasis was made by parasite detection or dramatic response to empirical therapy with an anti-parasitic agent (albendazole or ivermectin). Parasites could be detected by either stool examination or small intestinal tissue sample. Dramatic response was defined by improvement of symptoms within 2 weeks and an increase in serum albumin level to within normal limit within 2 months after receiving anti-parasitic agent. We collected 1) baseline characteristic data including age, sex, rural, urban or both living area, history of raw fish ingestion; 2) clinical presentation including chief complaint, duration of symptoms, details of each symptom; 3) laboratory tests including CBC, blood biochemistries, stool examination; 4) endoscopic data including type of endoscopic technique and their descriptive findings; and 5) small bowel radiologic findings which were reviewed and described by gastrointestinal radiologist (Apisarnthanarak P).

All of the data were recorded and analyzed using PASW Statistics for Windows, Version 18.0 (Chicago: SPSS Inc.). The results were summarized using descriptive statistics and Kaplan-Meier Curve was used for estimating a cumulative probability of improvement after treatment over time.

## Results

Of the 26 patients (Table 1), 20 (77%) were male; the gender ratio between male and female was 3.3:1. Patient age ranged from 18 to 71 years with a mean age of 43. Seventeen patients (65%) lived in rural area. History of raw fish consumption was available in 15 patients; of these, 14 patients (93%) admitted consuming it.

**Table 1:** **Baseline characteristics and clinical manifestations**

**Characteristics**	**n**	
Male gender, n (%)	20	(77)
Patients’ habitat, n (%)		
Urban	8	(31)
Rural	17	(65)
Both	1	(4)
Ingestion of raw fish, n (%)	14/15	(93)
Median duration of symptoms, month (range)	5.5	(1-60)
Less than 2 wk	0	
2 wk - 2 months	1	(4)
> 2-6 months	14	(54)
> 6-12 months	6	(23)
More than 12 months	5	(19)
Chief complaint, n (%)		
Chronic diarrhea	20/26	(77)
Abdominal pain	3/26	(11.5)
Edema	3/26	(11.5)
Chronic diarrhea, n (%)	24/26	(93)
Abdominal pain, n (%)	18/26	(70)
Edema, n (%)	25/26	(97)
Anemia, n (%)	13/26	(50)
Weight loss, n (%)	23/25	(92)
Median weight loss (range)	13 kg	(4-27)
Median % of weight loss from baseline (range)	23%	(10-40)
Features of generalized malabsorption, n (%)	14/24	(59)
Borborygmi, n (%)	10/11	(91)

### Clinical manifestation

Chronic diarrhea was the most common chief complaint (77%) while chronic abdominal pain and edema were less common (11.5% each). The median duration of symptoms before diagnosis was 5.5 months (ranging from 1 to 60 months). Five (19%) patients had symptoms of more than one year before diagnosis. The details of clinical manifestations are demonstrated in Table 1.

#### Chronic diarrhea

The stool character was watery in 21 patients (88%) and steatorrhea in 3 patients (12%). The frequency of stool per day varied among patients with a range of 0 to 20 times, with most of the patients passing stool 4 to 6 times. The course of chronic diarrhea was continuous in 16 (75%) and intermittent in 5 patients (25%). Two patients had no diarrhea and presented with severe abdominal pain and generalized edema.

#### Chronic abdominal pain

Abdominal pain was not localized in any parts of abdomen. The pain character was colicky in 13 of 18 patients (76%) and the course was continuous in 12 patients (67%). Abdominal borborygmi was present in 10 of 11 patients whose records were available.

### Laboratory results

All patients had hypoalbuminemia (mean 1.4 g/dl, range 0.6 to 3 g/dL), and approximately 90% had an albumin level of less than 2.5 g/dL. Nearly 70% (18/26) of patients had hypokalemia. Thirteen cases (50%) were anemic. Two cases (8%) had an absolute eosinophil of more than 500 /mm^3^. Other laboratory results are shown in Table 2.

**Table 2:** **Laboratory results**

**Parameters**	**Value**	
Hb, mean (SD)	11.4	(2.5)
Hb < 10 g/dl – n (%)	8/26	(30)
Eosinophilia (> 500 /mm^3^) – n (%)	2/26	(8)
K, mean (SD)	3.0	(0.9)
Albumin, mean (SD)	1.4	(0.5)
Less than 2 g/dL, n (%)	22	(85)
2-3.5 g/dL, n (%)	4	(15)
Cholesterol, mean (SD)	96.3	(24.7)
Iron deficiency anemia, n (%)	1/14	(7.1)
Folate deficiency, n (%)	3/13	(23.1)
B 12 deficiency, n (%)	2/12	(16.7)
Positive stool WBC, n (%)	4/26	(15.4)
Positive stool fat, n (%)	9/16	(56.3)

### Stool exam

Stool examinations revealed *Capillaria philippinnensis* ova in 15 patients (57.7%), of which 3 patients were discovered to have ova during bowel preparation for endoscopy. Other parasites were coincidentally found in 8 patients. They were *Opisthorchis viverrini* in 5 patients, *Strongyloides stercoralis* in 2 patients, and hookworm in 1 patient. The number of stool collections before diagnosis varied from 1 to 9 with a median of 4. Qualitative stool fat was positive in 9 of 16 patients (56%).

### Endoscopy

**Figure 1:**
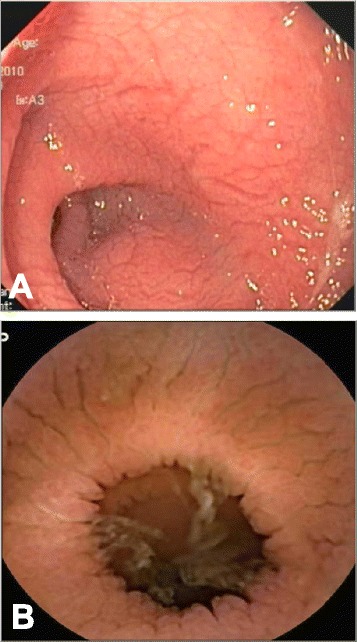
**Endoscopic findings. A**. Enteroscopic findings **B**. Capsule endoscopic findings.

**Figure 2:**
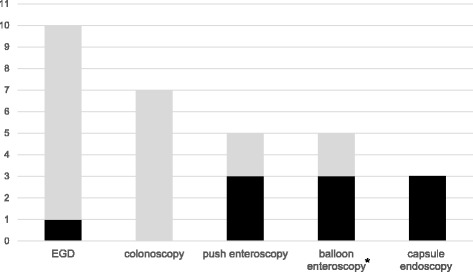
Yield of each endoscopy for diagnosis of intestinal capillariasis (*The negative findings of balloon enteroscopy in two patients were attributed to failure to reach the affected part).

EGD was performed in 10 patients; minor abnormalities of duodenal mucosa, including edematous and erythematous mucosa, was observed in 2 patients, but the parasite was noted in only 1 patient (sensitivity 10%). Colonoscopy was performed in 7 patients with swelling of ileum mucosa noted in 1 patient; however, the parasite was not found from biopsy specimen. Push enteroscopy was performed in 5 patients whose EGD findings were normal; obvious mucosal abnormalities, including diffuse flattening of villi causing a “scalloping appearance”, mucosal cracking, and erythematous mucosa (Figure 1), were observed in 3 patients. These 3 patients had parasite found in their biopsy samples. The other 2 patients underwent single balloon enteroscopy afterwards, and parasites were discovered in the more distal parts of small bowel. Thus, the sensitivity of push enteroscopy was 60%. Five patients underwent single balloon enteroscopy. Abnormalities of mucosa as seen in push enteroscopy were noted in 3 patients, and parasites were found from their biopsy samples. The negative findings of the balloon enteroscopies in the other two patients were probably attributable to the failure to reach the affected part – as one patient had abnormal small bowel mucosa on VCE, and the other had long segmental thickening of small bowel on CT abdomen. VCE was performed in 3 patients, all of which showed obvious small bowel mucosal lesions (Figure 1). Yield of each endoscopy for diagnosis of intestinal capillariasis was summarized in Figure 2.

Regarding endoscopic findings, the disease could involve duodenum, jejunum or ileum; however, the abnormalities were found most intense in jejunum and proximal ileum. The findings from endoscopy mimicked endoscopic findings described in celiac disease except the duodenal involvement, which is more common in celiac disease.

### Radiology

**Figure 3:**
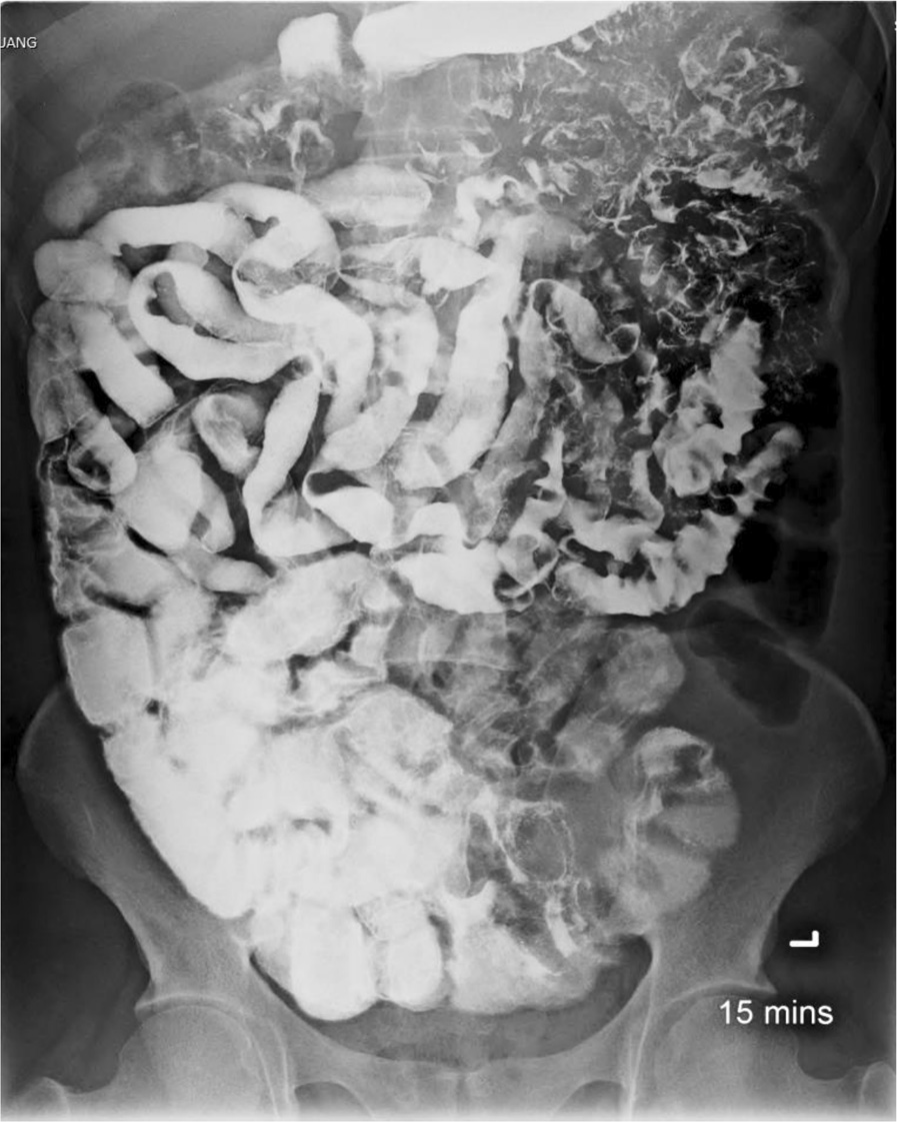
**Small bowel follow through study in a patient with intestinal capillariasis.**

Ten patients underwent small bowel barium study. Common findings included continuous long segment of fold thickening (90%), fold effacement (80%), and increased luminal fluid (90%) (Figure 3). Other findings were luminal narrowing (60%), luminal dilatation (10%), and flocculation of contrast (20%). Most patients had rapid bowel transit. The disease mostly involved distal jejunum and ileum. Only 2 patients had radiologic abnormalities in the duodenum.

Two patients underwent abdominal CT scans, showing continuous long segment of thickened and enhanced wall with fold effacement predominantly at the distal jejunum and ileum, along with mesenteric lymphadenopathy. One patient had mural cystic lesions and calcification prominent at the distal jejunum; such findings has never been described in intestinal capillariasis. Whether it has any causal relationship is not known.

### Diagnostic method

Twelve patients were solely diagnosed based on the presence of *C. phillippinensis* in stool. Three patients had *C. phillippinensis* discovered in both stool examination and tissue biopsies from their small bowels (2 by push enteroscopy, 1 by BAE). Four patients were solely diagnosed by histopathological finding of *C.phillippinensis* (1 by EGD, 1 by push enteroscopy, and 2 by BAE - one by the antegrade route and the other by the retrograde route). The remaining 7 patients who did not have parasite found were diagnosed from compatible clinical, radiologic and endoscopic findings together with dramatic response to therapeutic trial with either albendazole or ivermectin. The diagnostic methods were summarized in Table 3.

**Table 3:** **Diagnostic method**

**Diagnostic method**	**n**	
Stool examination, n (%)	15	(57)
No. of stool exam, median (range)	4	(1-9)
Histopathology, n (%)	4	(16)
EGD	1	(4)
Push enteroscopy	1	(4)
Single balloon enteroscopy	2	(8)
Empirical treatment, n (%)	7	(27)

### Treatment and response

Twenty patients were treated with albendazole at the dose ranging from 400-800 mg/d for 10-45 days. One patient was treated with 400 mg/day of mebendazole for 30 days. Six patients were treated with ivermectin at a dose of 12 mg/day for 3-10 days. All patients responded well to treatment except one patient who relapsed after treatment with 800 mg/day of albendazole for 21 days. In addition, he had another relapse despite treatment with 12 mg/day of ivermectin for 3 days. He was later proved to have reinfection rather than relapsed because of returning to eating raw fish.

**Figure 4:**
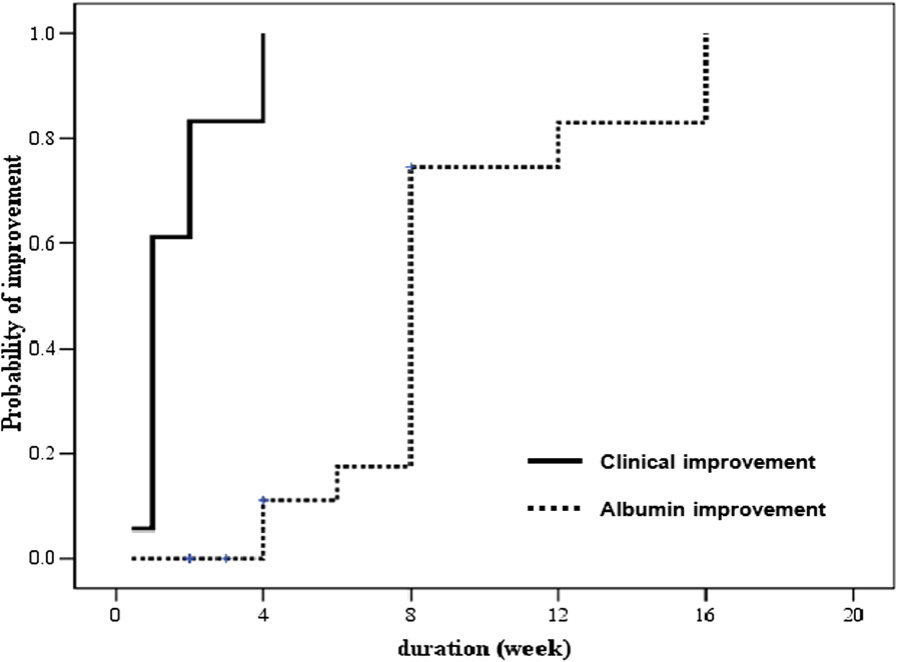
**Kaplan-Meier analysis of the cumulative probability of improvement after treatment over time.**

Figure 4 demonstrates the cumulative probabilities of clinical and laboratory (serum albumin) improvement over time after anti-parasitic treatment. The median improvement times of clinical and serum albumin were 1 month and 2 months, respectively.

## Discussion

Despite having been known for a long time, intestinal capillariasis is still problematic in clinical practice, especially in endemic area. To the best of our knowledge, this is the first study describing intestinal capillariasis in depth including the clinical manifestations and the findings of endoscopy and imaging using the more recently available technology. We also did a mini systematic review including previous case series which had at least 10 cases to compare with our data as shown in Table 4.

**Table 4:** **Published case series of intestinal capillariasis**

**Baseline characteristics**	**Present study**	**Kunaratanapruk, 1983** **[** 10 **]**	**Chunlertrith, 1992** **[** 8 **]**	**Bair, 2004** **[** 18 **]**	**Lu, 2006** **[** 17 **]**	**Thewjitcharoen, 2012** **[** 9 **]**	**Attia, 2012** **[** 19 **]**	**Total**
Number of patients	26	58	17	14	30	10	21	176
Age (yrs) - Mean (range)	42.6 (18-71)	8 – 63	40.4 (21-69)	52 (39-76)	50 (12-76)	37.7 (11-59)	32.7 (9-50)	
Male	20 (77)	40 (69)	13 (76)	9 (64)	18 (60)	6 (60)	2 (10)	108 (61)
Raw fish ingestion	14/15 (93)	NR	NR	NR	16 (53)	9 (90)	NR	39/55 (71)
Median duration of symptoms, mo (range)	5.5 (1 m - 5 y)	NR (1 d – 14 m)	7 (1 m – 3 y)	2.5 (1 wk – 31 m)	3 (1 wk – 6 y)	5 (4 d – 2 y)	7 (3 – 18 m)	1 d-72 m
Duration > 12 mo	5 (19)	NR	3 (18)	2 (15)	5 (17)	2 (20)	2 (10)	19 (11)
Chronic diarrhea	24/26 (93)	57/58 (98)	17/17 (100)	14/14 (100)	NR	10/10 (100)	21/21 (100)	143/146 (98)
Abdominal pain	18/26 (70)	32/58 (56)	16/17 (94)	12/14 (85)	NR	NR	NR	78/115 (68)
Weight loss	23/25 (92)	57/58 (98)	17/17 (100)	11/14 (78)	NR	9 (90)	NR	117/125 (93)
Hypoalbuminemia	26/26 (100)	NR	16/16 (100)	13/14 (93)	30/30 (100)	10/10 (100)	21/21 (100)	116/117 (99)
Albumin < 2 g/dL	22/26 (85)	NR	12/16 (75)	NR	19/30 (63)	9/10 (90)	17/21 (81)	79/103 (77)
Anemia	13/26 (50)	Uncommon	8/17 (47)	9/14 (64)	NR	NR	7/21 (33)	37/78 (47)
Eosinophilia	2/26 (8)	13 (22) (E > 10%)	NR	NR	11 (36) (E > 5%)	NR	0 (E > 10%)	
Presence of parasite in stool	15 (57)	58 (100)	17 (100)	10 (71.4)	21 (70)	9 (90)	Majority of patients	

Data presented are number of patients unless specified. Data in the parentheses are percentages.

*Capillaria philippinensis* is transmitted to humans by their consumption of raw fresh-water fish containing its larvae. Therefore, in this study, it affected people in rural areas of Thailand, who habitually eat raw fresh-water fish more than people in urban areas. However, nearly 30% of the patients had been living in an urban area for more than 5 years before diagnosis. This might be attributable to the increasing rate of immigration from rural to urban areas in Thailand. History of eating raw fresh-water fish was important because we found it in about 90% of patients. Nonetheless, it is not necessary to be present because many patients might not be able to recall it; nearly 50% of patients in the study by Lu et al. did not have this history (Table 4) [17].

Patients commonly presented with a duration of symptoms around 2 to 12 months; however, about 20% of patients in this study suffered from symptoms of more than one year and one patient for 5 years. The duration of symptoms in this series is similar to other reports [8-10,17-19] (Table 4), reflecting that intestinal capillariasis is difficult to diagnose and needs early recognition. Typically, the patients in our study presented with chronic watery diarrhea, which was not always continuous, accompanied by significant weight loss and protein losing enteropathy. However, some patients presented with other symptoms apart from chronic diarrhea such as chronic severe abdominal pain or generalized edema without diarrhea as shown in 2 patients in this report. Weight loss and severe hypoalbuminemia were very important features since more than 90% of patients had severe weight loss and about 80% had an albumin level lower than 2 g/dL. Although intestinal capillariasis mostly involves the long segment of jejunum and ileum, the clinical features of generalized malabsorption except edema were found in only 60% of patients. In particular, anemia was present in only 50% of patients. Eosinophilia was uncommon (less than 10% of patients) probably because *Capillaria philippinensis* is a less invasive tissue parasite compared to other invasive parasites such as *Strongyloides stercoralis*.

The diagnostic method of intestinal capillariasis in this study was somewhat different from those published before 2000, of which diagnoses all were made by discovering the parasites in stool (Table 4). Stool examination played a role in diagnosis in only about 60% of our patients, and most of the patients needed several stool examinations on different occasions. This confirms the low sensitivity of stool examination probably due to the low parasite load and intermittent shedding of the parasite into stool. In this study, we showed the higher sensitivity of tissue biopsy at the affected jejunum and ileum. In patients who were performed enteroscopies (push and balloon-assisted enteroscopy), the parasites were 100% found if the tissue samples were taken from obviously abnormal small bowel mucosa. Thus, this method appears to be one of the promising options to obtain the diagnosis, especially if the stool examination results are negative. However, we failed to reach the affected parts of small bowel in some patients due to wrong selection of endoscopic technique. Therefore, mapping the abnormal segment may be performed by imaging, either barium study or CT scan to help the selection of the enteroscopic route. The findings of scallop appearance of mucosa reflecting villous atrophy and erythematous and edematous mucosa from mucosal inflammation were homogenous in all patients. These findings were similar to the previously described findings by Rana et al. [12] and were also indistinguishable from celiac disease except for the involvement of duodenum, which was more prominent in celiac disease.

Interestingly, we observed that *C. philippinensis* was discovered in stool during bowel preparation for endoscopy in 3 of 15 patients. In these 3 patients, the stool examination results were negative several times before the bowel preparation. Theoretically, stool collection during bowel preparation might increase sensitivity owing to the increase in numbers of parasites shedding into stool.

The small bowel barium study findings of intestinal capillariasis have recently been described by Apisarnthanarak P et al. [20]. They reported the findings of continuous long segments of small bowel luminal narrowing with fold effacement and fold thickenings, mainly found at distal jejunum and proximal ileum. In our study, similar findings were found in most patients.

Response to treatment was very good in this study. Either an adequate dose of albendazole or ivermectin could cure all patients without any side effects. The relapse in 1 patient was because of his return to consumption of raw fish. All patients obtained clinical improvement within a month and 75% of patients had normal albumin levels within 2 months. Indeed, the response time might have been quicker if patients had been followed at closer time intervals. In this point, this study supports the role of empirical treatment in the area with high prevalence of intestinal capillariasis and very low prevalence of celiac disease, especially if enteroscopy or VCE are not available.

The limitation of this study is that it was the retrospective study and had a somewhat small number of patients. However, because the results were concordant with previous studies, this confirms that the data should be reliable and can be applicable for use in clinical practice. Another possible limitation is the possibility that response to empirical treatment may not definitely confirm the diagnosis of intestinal capillariasis. Other parasites, especially strongyloidiasis in hyperinfection form, may have presented a similar clinical picture and response to albendazole or ivermectin. Nonetheless, strongyloid hyperinfection usually occur in patients who use corticosteroid. None of the patients in our study were taking it.

## Conclusions

**Figure 5:**
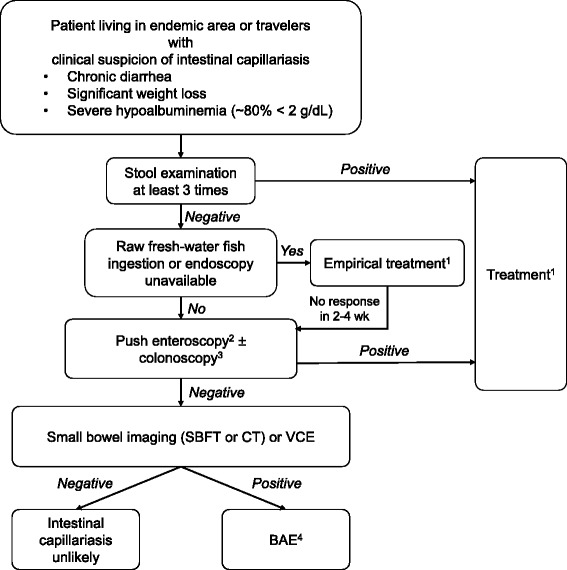
**Proposed algorithm for diagnosis of intestinal capillariasis.** ^1^Treatment regimen: albendazole 400 mg/day for 10 days. ^2^Push enteroscopy is preferred to EGD due to its higher diagnostic yield and its similar availability to colonoscopy. ^3^Colonoscopy if push enteroscopy findings are negative. ^4^Route of BAE is guided by imaging or VCE findings. Abbreviations: *SBFT* small bowel follow through, *VCE* video capsule endoscopy, *BAE* balloon assisted enteroscopy, *EGD* esophagogastroduodenoscopy.

In an endemic area, one should suspect intestinal capillariasis in patients presenting with chronic watery diarrhea accompanied by significant weight loss and severe hypoalbuminemia. However, one should be aware that some patients may present with chronic abdominal pain without diarrhea. Stool examination has limited sensitivity; therefore, even if the result is negative, enteroscopy should be considered. Stool collection during bowel preparation could be another option because it may increase the sensitivity. Empirical therapy should be considered when endoscopy is not feasible. A proposed algorithm for diagnosis of intestinal capillariasis is shown in Figure 5.
